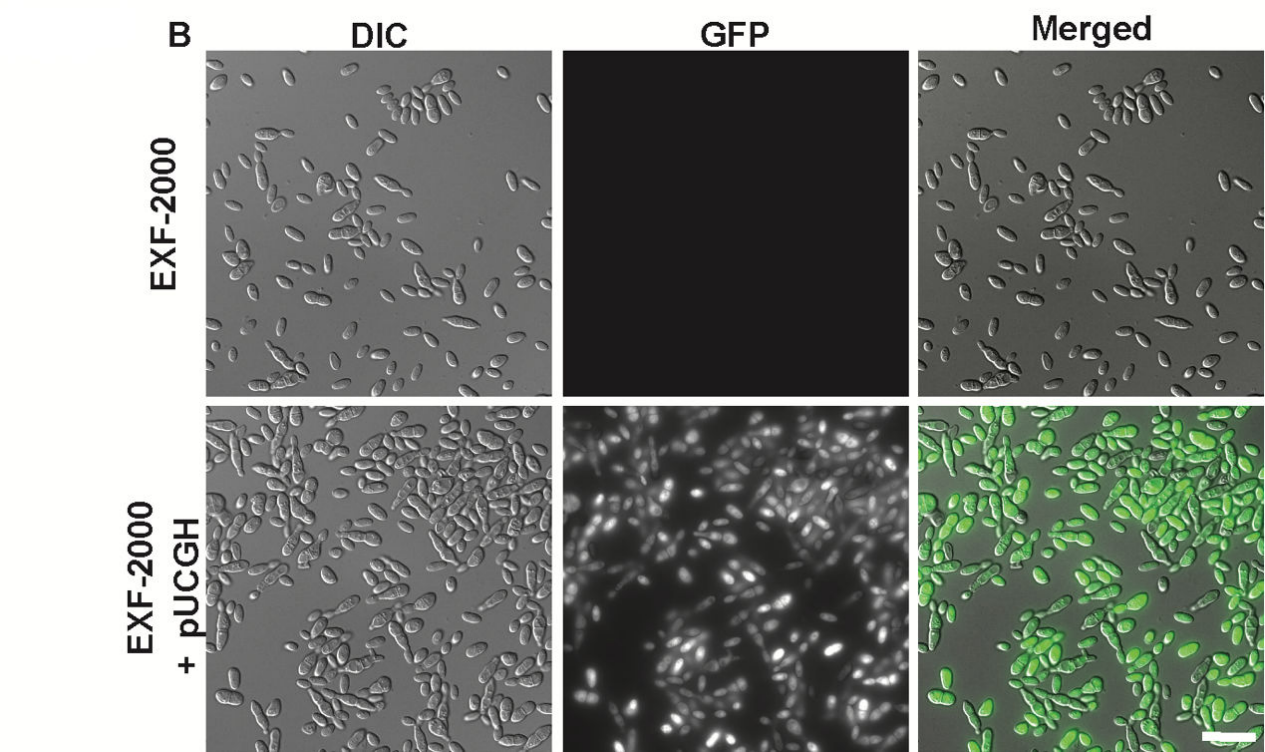# Erratum for Hernandez-Rodriguez et al., “Strategies for genetic manipulation of the halotolerant black yeast *Hortaea werneckii*: ectopic DNA integration and marker-free CRISPR/Cas9 transformation”

**DOI:** 10.1128/spectrum.00338-25

**Published:** 2025-04-04

**Authors:** Yainitza Hernandez-Rodriguez, A. Makenzie Bullard, Rebecca J. Busch, Aidan Marshall, José M. Vargas-Muñiz

## AUTHOR CORRECTION

Volume 13, no. 1, 13, e02430-24, 2025, https://doi.org/10.1128/spectrum.02430-24. Page 5, Fig. 1B: The DIC panel for the EXF-2000 + pUCGH strain was incorrectly duplicated. Fig. 1B was updated to contain the correct DIC of the EXF-2000 + pUCGH strain.

**Fig 1:**